# Enhancing detection of high-level axillary lymph node metastasis after neoadjuvant therapy in breast cancer patients with nodal involvement: a combined approach of axilla ultrasound and breast elastography

**DOI:** 10.1007/s11547-024-01936-2

**Published:** 2024-11-20

**Authors:** Jia-Xin Huang, Feng-Tao Liu, Yu-Ting Tan, Xue-Yan Wang, Jia-Hui Huang, Shi-Yang Lin, Gui-Ling Huang, Yu-Ting Zhang, Xiao-Qing Pei

**Affiliations:** 1 Department of Liver Surgery, State Key Laboratory of Oncology in South China, Guangdong Provincial Clinical Research Center for Cancer, Collaborative Innovation Center for Cancer Medicine, Sun Yat-Sen University Cancer Center, Guangzhou, 510060 People’s Republic of China; 2 Department of Medical Ultrasound, State Key Laboratory of Oncology in South China, Guangdong Provincial Clinical Research Center for Cancer, Collaborative Innovation Center for Cancer Medicine, Sun Yat-Sen University Cancer Center, Guangzhou, 510060 People’s Republic of China; 3 Breast Tumor Center, Sun Yat-Sen Memorial Hospital, Sun Yat-Sen University, Guangzhou, 510000 People’s Republic of China; 4 Institute of Artificial Intelligence and Blockchain, Guangzhou University, Guangzhou, 510000 People’s Republic of China; 5 Department of Medical Ultrasound, The Sixth Affiliated Hospital of Sun Yat-Sen University, Guangzhou, 510000 People’s Republic of China

**Keywords:** Axilla, Breast neoplasm, Neoadjuvant therapy, Elastography, Ultrasound

## Abstract

**Purpose:**

To develop a combined approach using shear wave elastography (SWE) and conventional ultrasound (US) to determine the extent of positive axillary lymph nodes (LNs) following neoadjuvant therapy (NAT) in breast cancer patients with nodal involvement.

**Methods:**

This prospective, multicenter study was registered on the Chinese Clinical Trial Registry (ChiCTR2400085035). From October 2018 to February 2024, a total of 303 breast cancer patients with biopsy-proven positive LN were enrolled. The conventional US features of axillary LNs and SWE characteristics of breast lesions after NAT were analyzed. The diagnostic performances of axilla US, breast SWE, and their combination in detecting residual metastasis in axillary level III after NAT were assessed.

**Results:**

Pathologically positive LN(s) in axilla level III were detected in 13.75% of cases following NAT. The kappa value for the axilla level with positive LN confirmed by surgical pathology and detected by US is 0.39 (*p* < 0.001). The AUC of conventional axilla US to determine the status of axilla level III LNs after NAT was 0.67, with a sensitivity of 51.52%, a specificity of 74.36%. The breast SWE displayed moderate performance for detecting residual metastasis in axilla level III following NAT, with an AUC of 0.79, sensitivity of 84.85%, and specificity of 74.36%. Compared to axilla US and breast SWE alone, the combination of axilla US with breast SWE achieved a stronger discriminatory ability (AUC, 0.86 vs 0.67 vs 0.79, *p* < 0.05, Delong’s test) and precise calibration (*X*^2^ = 13.90, *p* = 0.085, HL test), with an improved sensitivity of 93.94% and a comparable specificity of 75.64%%.

**Conclusions:**

SWE outperformed conventional US in identifying the axilla levels with nodal metastasis following NAT in patients with initially diagnosed positive axilla. Furthermore, combining breast SWE with axilla US showed good diagnostic performance for detecting residual metastasis in axilla level III after NAT.

## Introduction

Neoadjuvant therapy (NAT) has been proved as a common treatment for breast cancer patients with pathologically confirmed lymph node (LN) metastasis, offering advantages of reducing the tumor burden in both breast and axillary LNs [1]. And patients with a reduced tumor burden after NAT are increasingly being considered for less radical surgical treatments. In order to prevent overtreatment of the axilla after NAT, breast cancer patients who exhibit a significant response to NAT may consider less invasive axillary procedures as an alternative to axillary lymph node dissection (ALND) [2]. However, there is currently no consensus on the appropriate selection criteria for such approaches. ALND has traditionally served as the standard restaging procedure for axillary LNs after NAT in patients with confirmed node-positive breast cancer, especially in clinical N2-3 patients [2, 3].

The standard extent of ALND in patients with node-positive breast cancer involves clearing axilla level I and level II LNs, while the clearance of axilla level III LNs has been a subject of debate [4]. It has been observed that 9.0–15.5% of breast cancer cases with nodal involvement exhibit positivity of axilla level III LNs following NAT [5, 6]. The presence of positive axilla level III LNs is considered a risk factor for distant metastasis and tumor recurrence [4]. Generally, clearance of axilla level III LNs is considered when clinical assessment indicates extensive nodal involvement in the axilla and/or significant involvement of axilla levels II/III LNs [7, 8]. However, the value and indications of clearing axilla level III LNs are limited by the accuracy of clinical assessment for this specific axilla region [4]. Preoperatively defining the axilla level with metastatic LNs holds important reference value for guiding the extent of ALND surgery. Therefore, radiologists have increasingly directed their focus toward excluding high-level axillary LN metastasis and advanced LN metastasis, instead of solely concentrating on detecting individual LN metastasis [9, 10]. Nevertheless, accurately predicting axillary pathologic complete response (pCR) remains a significant difficulty in patients with node-positive breast cancer, and determining the extent of positive LNs in the axilla after NAT is particularly challenging.

Ultrasound (US) is the preferred imaging approach for assessing axillary LNs after NAT in breast cancer patients [11]. However, in patients with nodal involvement at initial diagnosis, conventional US has limitations in determining axillary status following NAT [12]. As a relatively new US technology, ultrasonic elastography (UE) has shown promise in predicting breast tumor response to NAT and assessing the nodal status in breast cancer patients [13–18]. Nevertheless, the specific role of UE in predicting nodal response to NAT and determining the extent of positive axillary LNs remains unclear.

In order to permit less extensive surgery of axilla following NAT, this study was designed to explore the added value of shear wave elastography (SWE) in determining the axilla level with positive LNs compared to conventional axilla US. Furthermore, it aimed to investigate whether the combination of conventional US with SWE can accurately detect residual metastasis in axilla level III after NAT in breast cancer patients with nodal involvement.

## Methods

### Ethics

This prospective study was registered on the Chinese Clinical Trial Registry (ChiCTR2400085035) and approved by the institutional review board's ethics committee (B2022-373-X01). Written informed consent was obtained from all patients.

### Patients

**Fig. 1:**
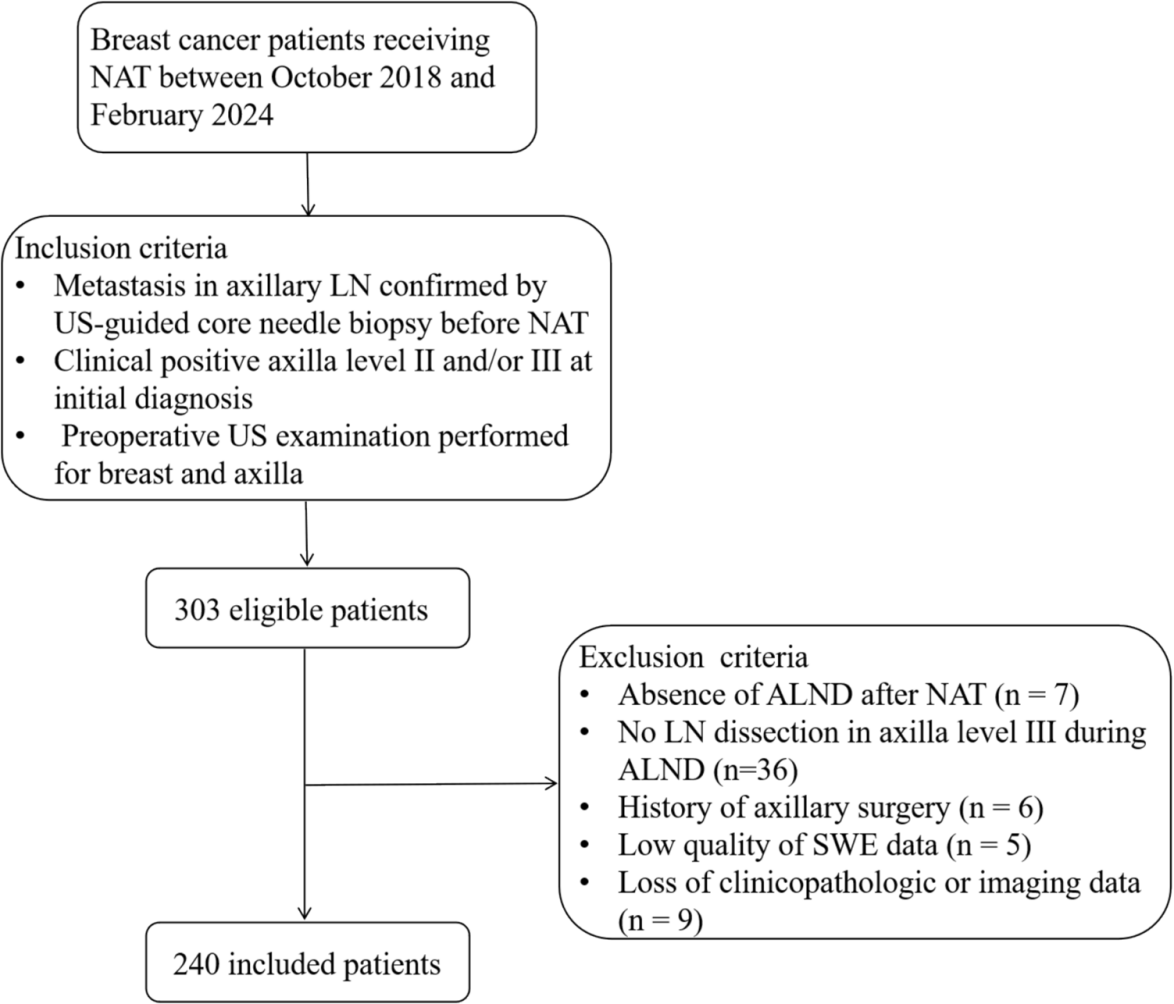
Flowchart of the study population. NAT, neoadjuvant therapy; LN, lymph node; US, ultrasound; ALND, axillary lymph node dissection; SWE, shear wave elastography

This prospective study was conducted on 303 patients with node-positive breast cancer at Sun Yat-Sen University Cancer Center and Sun Yat-sen Memorial Hospital between October 2018 and February 2024. The inclusion criteria were: (i) metastasis in axillary LN confirmed by US-guided core needle biopsy before NAT; (ii) clinical positivity of axilla level II and/or III at initial diagnosis; and (iii) preoperative US examination performed for breast and axilla. 63 patients were excluded due to: (i) absence of ALND after NAT (*n* = 7), (ii) no LN dissection in axilla level III during ALND (*n* = 36), (iii) history of axillary surgery (*n* = 6), (iv) low quality of SWE data (*n* = 5), and (v) loss of clinicopathologic or imaging data (*n* = 9). Last, a total of 240 patients were included in this study, as shown in Fig. 1.

### US examinations

After the completion of NAT, patients received a US examination a day before surgery, including conventional US and SWE. The US examinations were performed using the Siemens S2000 ultrasound system equipped with a 7.0–12.0 MHz linear array transducer (Siemens Medical Solutions, Mountain View, CA, USA). First, the radiologists documented the conventional US characteristics of breast lesion according to BI-RADS lexicon. Subsequently, the US findings of axillary LN were recorded including the US features of suspicious LN and axilla level with suspicious LN. The suspicious US features of LN include ratio of long axis diameter to short axis diameter < 2, cortical thickening > 3 mm, rounded or irregular shape, effacement of the fatty hilum, presence of microcalcifications, replacement of the LN with an ill-defined or irregular mass, and nonhilar blood flow [19]. A positive axillary LN on US was defined if at least one suspicious US finding was identified. The axillary LNs were categorized into three levels (I, II, and III) based on their location within the lateral, medial, and posterior regions of the pectoralis minor muscle. Following the conventional US examination, SWE was conducted at the plane of the breast tumor's maximal diameter with the probe being held still, and patients being asked to suspend respiration for a few seconds. A quality map was obtained to assess the quality of the shear waves and reliability of the data acquisition, followed by the acquisition of a velocity map, with the shear wave velocity (SWV) values ranging from 0.5 to 10 m/s. Six measurement ROIs with a fixed size of 2 × 2 mm were placed on three areas with the highest stiffness and three areas with the softest stiffness within the breast lesion to obtain SWVmax and SWVmin values, respectively. The average value of SWV (SWVmean) was calculated for the six SWV-ROIs [18, 20, 21].

### Pathologic evaluation

Prior to treatment, the pathologic diagnosis of breast cancer and metastasis in axillary LNs was determined by a US-guided core needle biopsy. The biomarkers associated with molecular subtypes of breast cancer were identified through the use of immunohistochemistry and fluorescence in situ hybridization techniques. Following the completion of NAT, the included patients underwent mastectomy or breast-conserving surgery along with ALND. The number and location (axilla level) of pathologically positive LNs were recorded. A axillary pCR was defined as the absence of residual metastasis in all removed axillary LNs. Positive axilla level III was defined as at least one positive LN in axilla level III.

### Statistical analysis

Mean and standard deviation were employed to describe continuous data, whereas count was utilized for describing categorical variables. Continuous quantitative variables were compared between positive and negative axilla level III after NAT in patients with node-positive breast cancer, using either a t-test or Mann–Whitney U test. Univariate analysis of categorical variables was performed using either the χ2 test or Fisher's exact test. The k statistic was used to evaluate variability in US diagnosis and surgical result. The k value of 0.2 or less represented slight agreement, 0.21–0.40 indicated fair agreement, 0.41–0.60 signified moderate agreement, 0.61–0.80 denoted substantial agreement, and 0.81–0.99 represented almost perfect agreement. Receiver operating characteristic (ROC) curve analysis was employed to assess the discrimination of conventional axilla US and breast SWE in diagnosing LNs in axilla level III. A model was developed by combing axilla US and breast SWE findings through multivariable logistic regression (LR) analysis to predict the status of axilla level III after NAT in cases with nodal involvement. A calibration curve was applied to illustrate the correlation between the predicted and observed status of LNs in axilla level III. The clinical utility of the model was assessed through decision curve analysis. All statistical analyses were performed using SPSS version 25.0 and Medical version 19.0. All statistical tests were two-sided, and a *p* < 0.05 was considered statistically significant.

## Results

### Clinicopathologic characteristics

A total of 240 patients with node-positive breast cancer (mean age, 46.36 ± 10.23 years; range, 25–70 years) were included (Table 1). Among them, 129 (53.75%) cases achieved axillary pCR and 111 (46.25%) cases had residual metastasis in axillary LNs. Furthermore, 33 (13.75%) cases were found to have positive LN(s) in axilla level III while 207 (86.25%) cases had negative axilla level III after NAT. Patients with positive axilla level III after NAT were significantly more likely to have higher clinical tumor stage and nodal stage at initial, as well as negative HER2 expression. Table 1 presents the baseline clinicopathologic characteristics of the patients.

**Table 1:** Baseline clinicopathologic characteristics

Characteristics	All patients (*n* = 240)	Positive axilla level III		*p*-value
		No (*n* = 207)	Yes (*n* = 33)	
Age*, years	46.36 ± 10.23	45.95 ± 10.07	48.94 ± 10.99	0.119
Menopausal status, n (%)				0.732
Pre/perimenopausal	159	138 (86.8)	21 (13.2)	
Postmenopausal	81	69 (85.2)	12 (14.8)	
Tumor stage, n (%)				0.004
1	29	29 (100.0)	0 (0.0)	
2	128	107 (83.6)	21 (16.4)	
3	54	50 (92.6)	4 (7.4)	
4	29	21 (72.4)	8 (27.6)	
Nodal stage, n (%)				0.008
1	83	76 (91.6)	7 (8.4)	
2	82	74 (90.2)	8 (9.8)	
3	75	57 (76.0)	18 (24.0)	
Clinical stage, n (%)				0.121
2	54	50 (92.6)	4 (7.4)	
3	186	157 (84.4)	29 (15.6)	
ER expression, n (%)				0.538
Negative	69	61 (88.4)	8 (11.6)	
Positive	171	146 (85.4)	25 (14.6)	
PR expression, n (%)				0.104
Negative	104	94 (90.4)	10 (9.6)	
Positive	136	113 (83.1)	23 (16.9)	
HER2 expression, n (%)				< 0.001
Negative	120	90 (75.0)	30 (25.0)	
Positive	120	117 (97.5)	3 (2.5)	
Ki-67 score, n (%)				0.405
≤ 14%	39	32 (82.1)	7 (17.9)	
> 14%	201	175 (87.1)	26 (12.9)	

ER, estrogen receptor; PR, progesterone receptor; HER2, human epidermal growth factor receptor 2

*Data are means standard deviation

### Axilla US diagnosis

According to pathologic result of ALND, among 111 patients with axillary non-pCR, 47 (42.34%) cases had residual metastasis only in axilla level I; 31 (27.93%) cases had positive LNs in axilla level II and no positive LNs in axilla level III; positive LNs were found in axilla level III in 33 (29.73%) cases. Regarding axilla US diagnosis, no suspicious LNs were found in 120 cases, while 120 cases had positive LNs on US. Among the 120 cases with positive LNs on US, 73 cases were found to have positive LNs only in axilla level I, 36 cases in axilla level II, and 11 cases in axilla level III. The kappa value of axilla US diagnosis and ALND result for determining axilla level with positive LNs is 0.39 (*p* < 0.001), indicating a fair agreement (Table 2). According to ALND results, axilla US accurately identified 75.19% (97/129) of cases with axillary pCR, 61.70% (29/47) of cases with residual metastasis only in axilla level I, 35.48% (11/31) of cases in axilla level II, and 24.24% (8/33) of cases in axilla level III.

**Table 2:** Consistency between axilla US diagnosis and surgical-pathologic results of axillary levels with residual metastasis after NAT

Axilla level with positive LN		Surgical pathology				Total	*p-*value
		pCR	Level I	Level II	Level III		
US diagnosis	cCR	97	10	9	4	120	
	Level I	22	29	10	12	73	
	Level II	9	7	11	9	36	
	Level III	1	1	1	8	11	
Total		129	47	31	33	240	< 0.001

US, ultrasound; NAT, neoadjuvant therapy; pCR, pathologic complete response; cCR, clinic complete response

In this study, the negative/positive classification of axilla level III according to the ALND result served as the gold standard, while the axilla level with positive LNs diagnosed by US was considered as the independent variable. Based on ROC curve analysis, the performance of US diagnosis for LNs in axilla level III was found to be limited, with an area under ROC curve (AUC) of 0.67, an accuracy of 67.57%, a sensitivity of 51.52%, and a specificity of 74.36%. The cutoff value was determined to be a positive axilla level on US of > I, indicating that suspicious LNs in axilla level II or III on US were considered as predictors of pathologically positive axilla level III.

### Breast SWE performance

**Fig. 2:**
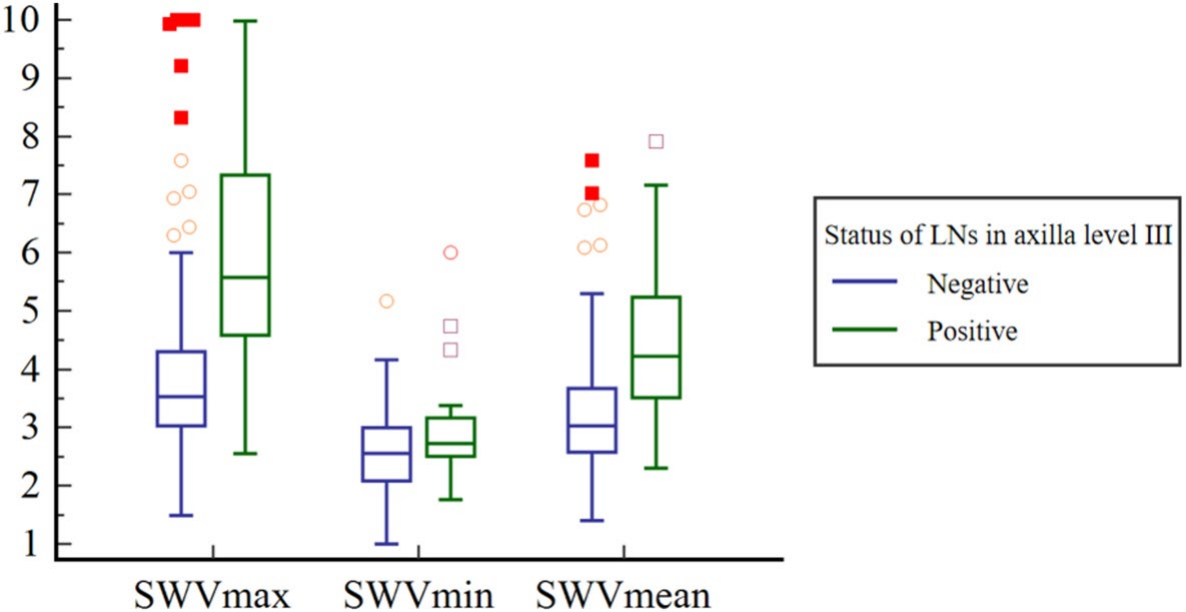
Clustered box plot of SWE characteristics for both negative and positive axilla level III after NAT. Abbreviations: SWE, shear wave elastography; NAT, neoadjuvant therapy; SWV, shear wave velocity; LN, lymph node

**Fig. 3:**
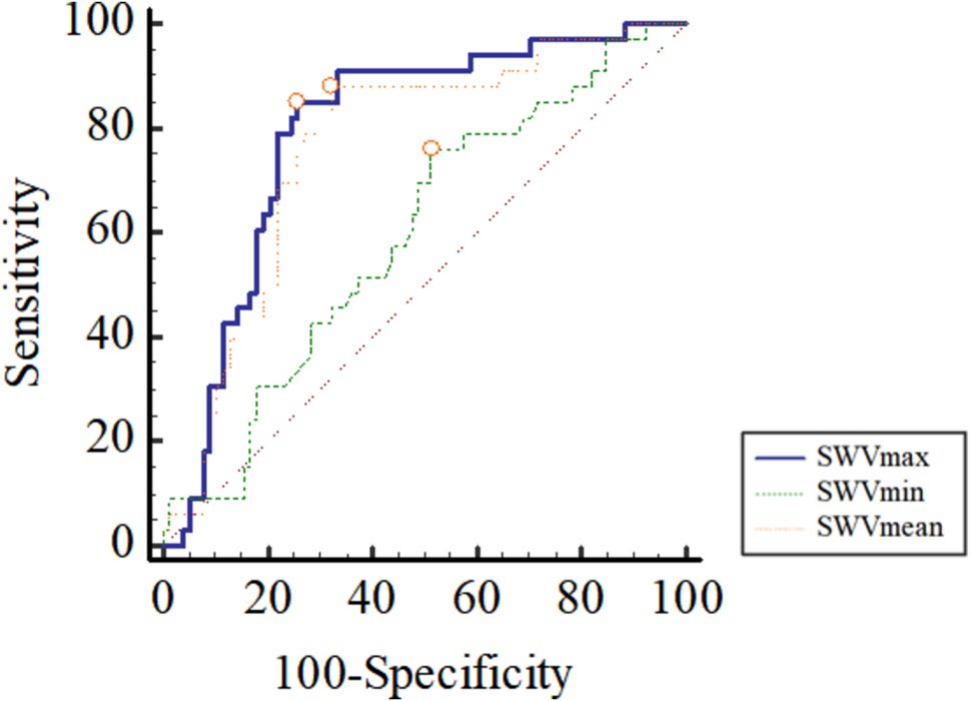
ROC curves for using SWE in diagnosing LNs in axilla level III after NAT in patients with node-positive breast cancer. Abbreviations: ROC, receiver operating characteristic; SWE, shear wave elastography; LN, lymph node; NAT, neoadjuvant therapy; SWV, shear wave velocity

For cases with negative axilla level III after NAT, the SWVmax, SWVmin, and SWVmean values of breast lesions were 3.18 ± 1.62 m/s, 2.21 ± 0.70 m/s, and 2.70 ± 1.11 m/s, respectively. In contrast, for breast lesions with positive axilla level III, the SWVmax, SWVmin, and SWVmean values were 5.97 ± 1.90 m/s, 2.89 ± 0.85 m/s, and 4.43 ± 1.27 m/s, respectively. All of these values were significantly higher than those for cases with negative axilla level III (*p* < 0.001), as indicated in Table 3 and Fig. 2. According to the ROC curve analysis, SWVmax exhibited the best performance in identifying positive axilla level III after NAT, with an AUC of 0.79. The optimal cutoff value of SWVmax was 4.21 m/s, with an accuracy of 77.48%, a sensitivity of 84.85%, and a specificity of 74.36% (Table 4). SWVmax demonstrated significantly superior performance for determining nodal status in axilla level III compared to SWVmin and SWVmean (SWVmax vs. SWVmin, *p* < 0.001 and SWVmax vs. SWVmean,* p* = 0.016, Delong’s test), as shown in Fig. 3.

**Table 3:** The comparison of breast SWE characteristics between negative and positive axilla level III groups

SWE characteristics	Total	Negative axilla level III	Positive axilla level III	*p*-value
SWVmax (m/s)	3.56 ± 1.92	3.18 ± 1.62	5.97 ± 1.90	< 0.001
SWVmin (m/s)	2.31 ± 0.76	2.21 ± 0.70	2.89 ± 0.85	< 0.001
SWVmean (m/s)	2.93 ± 1.28	2.70 ± 1.11	4.43 ± 1.27	< 0.001

SWE, shear wave elastography; SWV, shear wave velocity

**Table 4:** Diagnostic performance of SWE characteristics

SWE characteristics	AUC	Accuracy (%)	Sensitivity (%)	Specificity (%)	PPV (%)	NPV (%)	Youden index	Cut -off value	*p*-value
SWVmax (m/s)	0.79	77.48	84.85	74.36	58.33	92.06	0.59	> 4.21	< 0.001
SWVmin (m/s)	0.60	56.76	75.76	48.72	38.46	82.61s	0.25	> 2.51	< 0.001
SWVmean (m/s)	0.76	73.87	87.88	67.95	53.70	89.83	0.56	> 3.30	< 0.001

SWE, shear wave elastography; SWV, shear wave velocity; AUC, area under the receiver operating characteristic curve; PPV, positive predictive value; NPV, negative predictive value

### Combined evaluation

**Fig. 4:**
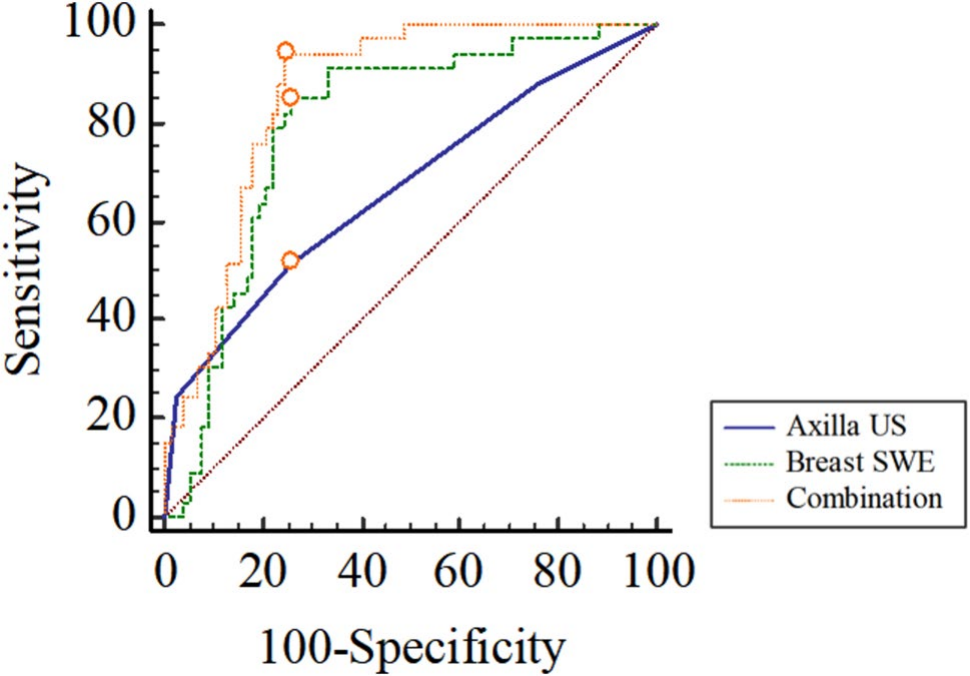
ROC curves for using SWE in determining the status of axilla level III after NAT in patients with node-positive breast cancer. Abbreviations: ROC, receiver operating characteristic; SWE, shear wave elastography; NAT, neoadjuvant therapy; US, ultrasound

In our study, axilla level with suspicious LNs on US and breast SWVmax were identified as associated variables with the pathologic status of axilla level III after NAT. A higher axilla level with positive LNs on US (OR, 1.36, 1.75, and 26.06 for positive axilla level I, II, and III on US, respectively) and a higher SWVmax of the breast lesions (OR, 1.58) were found to be independently associated with pathologically positive axilla level III. Consequently, a combined US model was developed based on axilla US diagnosis and breast SWE characteristics, using multivariate LR analysis. The combination of axilla level with suspicious LNs on US and the SWVmax of the breast lesion resulted in significantly higher performance than conventional axilla US alone (AUC, 0.86 vs 0.69, *p* < 0.001, Delong’s test) for identifying positive LNs in axilla level III after NAT, with an enhanced accuracy of 81.08%, a remarkably improved sensitivity of 93.94%, and a comparable specificity of 75.64%, at a predictive probability cutoff value of 0.18 (Table 5 and Fig. 4).

**Table 5:** The performance of axilla US, breast SWE, and their combination for detecting positive LNs in axilla level III after NAT in patients with node-positive breast cancer

US modalities	AUC	Accuracy (%)	Sensitivity (%)	Specificity (%)	PPV (%)	NPV (%)	Youden index	Cut -off value	*p-value*
Axilla US	0.67	67.57	51.52	74.36	45.95	78.38	0.26	> 1	< 0.001
Breast SWE	0.79	77.48	84.85	74.36	58.33	92.06	0.59	> 4.21	< 0.001
Combination	0.86	81.08	93.94	75.64	62.00	96.72	0.70	> 0.18	< 0.001

US, ultrasound; SWE, shear wave elastography; LN, lymph node; NAT, neoadjuvant therapy; AUC, area under the receiver operating characteristic curve; PPV, positive predictive value; NPV, negative predictive value

**Fig. 5:**
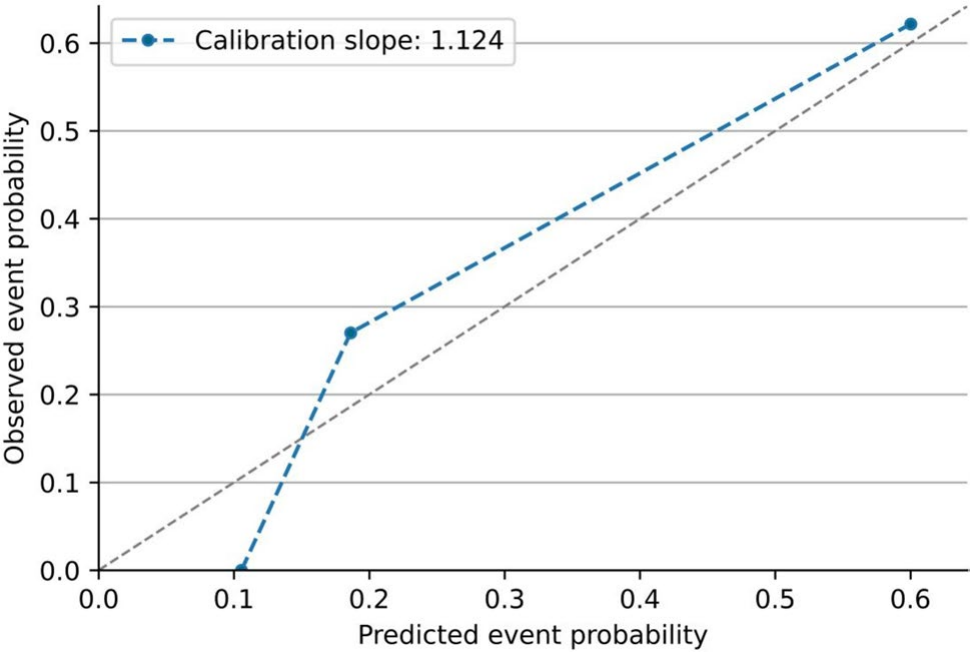
Calibration curve for the predictive model

**Fig. 6:**
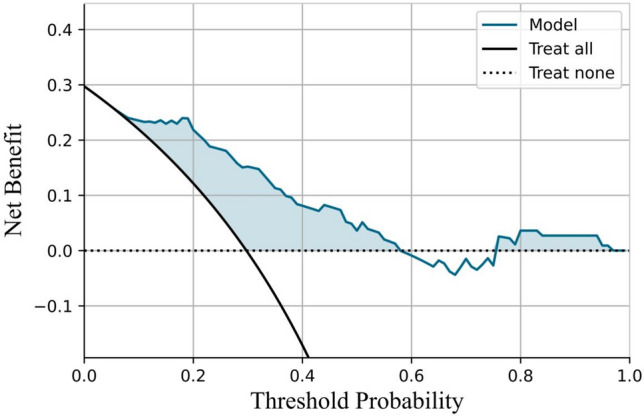
Decision curve for the clinical benefit of the predictive model

**Fig. 7:**
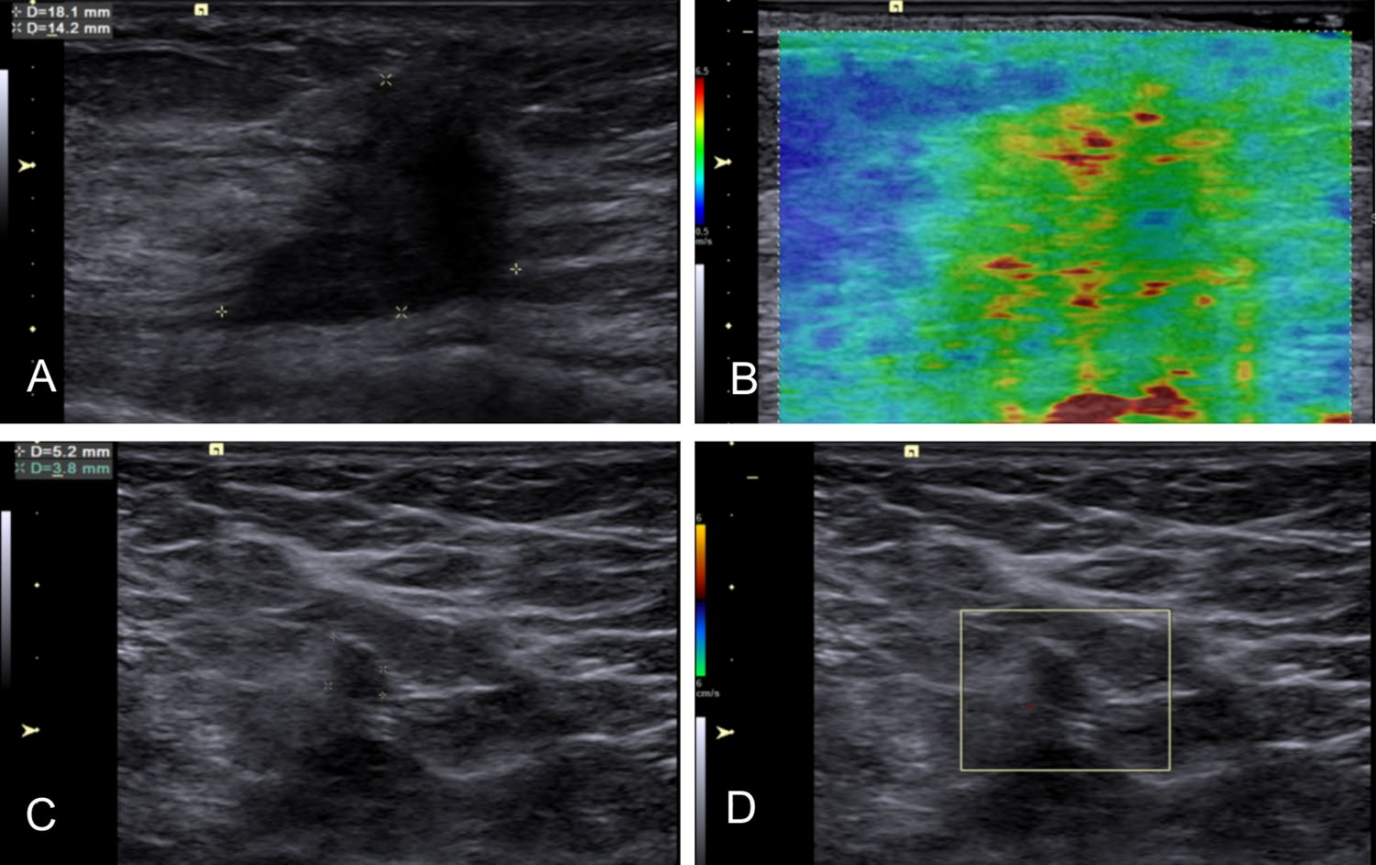
The characteristics of breast SWE and axilla US in a cases with positive axilla level III after NAT. A 53-year-old case with T2N3 Luminal-A breast cancer was found to have residual metastasis in 14 LNs in axilla level I, 3 in axilla level II, and 2 in axilla level III according to ALND result. **A** A solid, hypo-echogenic breast lesion measuring 18.1 × 4.2 mm was found on US. **B** The breast lesion showed a SWVmax value of 5.30 m/s on the SWE-velocity map. **C**–**D** Several suspicious small LNs were identified in axilla level I by using axilla US, characterized by ratio of long axis diameter to short axis diameter < 2, irregular shape, ill-defined margin, effacement of the fatty hilum, and absence of hilar blood flow. No LNs were detected in axilla level II or III on US. Based on the model combing breast SWVmax of 5.30 m/s and positive axilla level I on US, the predictive result indicated the presence of residual metastasis in axilla level III. SWE, shear wave elastography; US, ultrasound; NAT, neoadjuvant therapy; LN, lymph node; ALND, axillary lymph node dissection; SWV, shear wave velocity

**Fig. 8:**
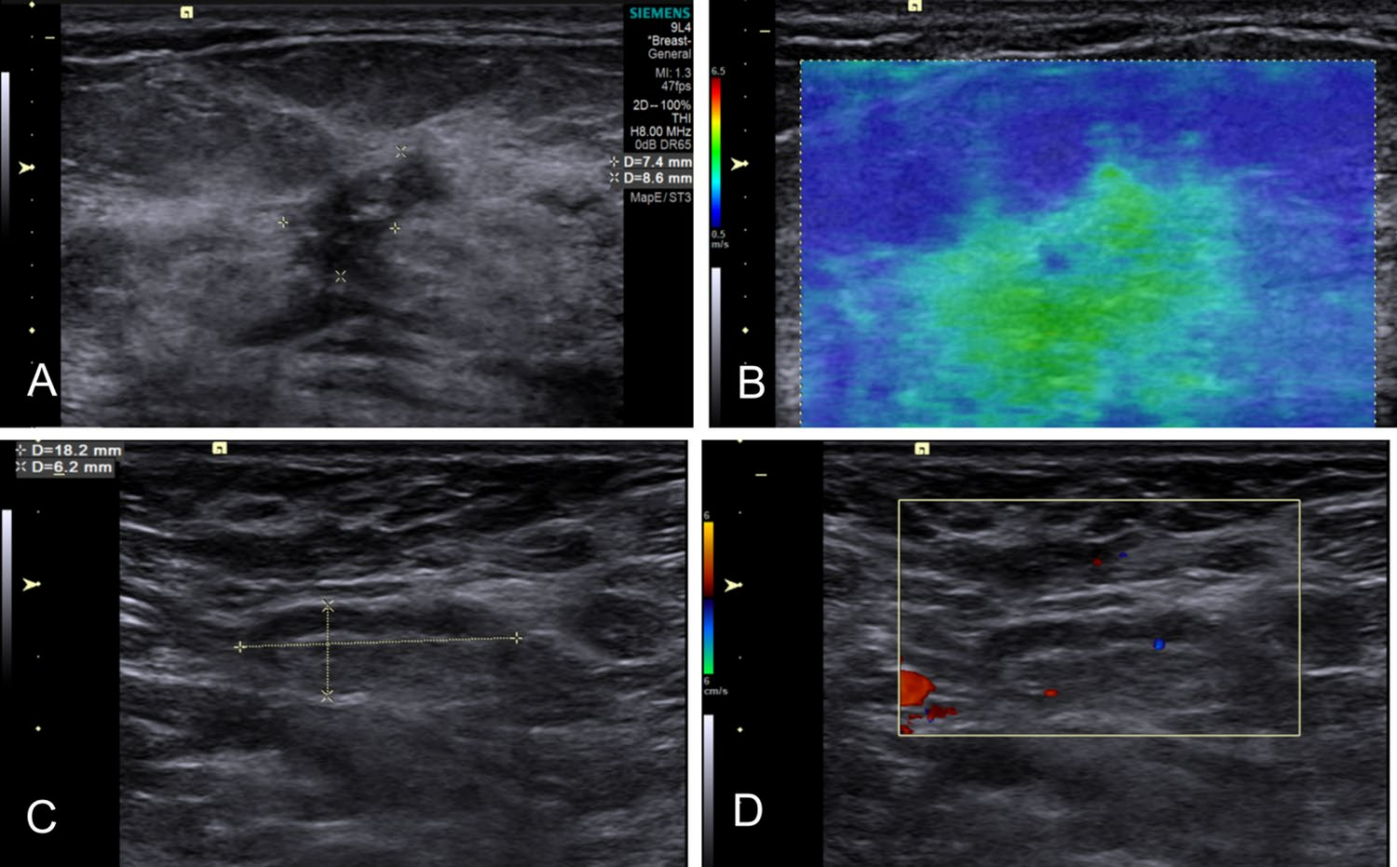
The characteristics of breast SWE and axilla US in a cases with negative axilla level III after NAT. Following NAT, a 50-year-old patient with T2N2 Luminal-B2 breast cancer underwent ALND, during which 21 lymph nodes were removed from axilla levels I-III. According to ALND result, only 1 LNs in axilla level II was found to have residual metastasis. **A** A solid, hypo-echogenic breast lesion with interior calcification measuring 8.6 × 7.4 mm was found on greyscale US. **B** The breast lesion showed a SWVmax value of 3.17 m/s on the SWE-velocity map. **C**–**D** Several LNs without suspicious features were identified in axilla level I, and no LNs were detected in axilla level II or III on US. Based on the model combing breast SWVmax of 3.17 m/s and negative axilla on US, the predictive result indicated the absence of residual metastasis in axilla level III. SWE, shear wave elastography; US, ultrasound; NAT, neoadjuvant therapy; ALND, axillary lymph node dissection; LN, lymph node; SWV, shear wave velocity

The *p*-value obtained using the Hosmer–Lemeshow goodness-of-fit test was 0.085 (*X*^2^ = 13.90), indicating a good fit of the combined model. Calibration curve analysis demonstrated good agreement between the observed and predictive status of LNs in axilla level III after NAT in breast cancer patients with positive LNs at the initial diagnosis (slope, 1.12), as depicted in Fig. 5. Additionally, the decision curve analysis revealed that, when the probability threshold was 0.00–0.57 and 0.75–0.96, clinical decision-making based on the combined model offered superior overall benefits compared to the all-or-none strategy, as depicted in Fig. 6. Figures 7 and 8 illustrate the effectiveness of this combined model.

## Discussion

Our study confirms that conventional axilla US alone is insufficient to determine the extent of positive axillary LNs after NAT in patients with node-positive breast cancer. Additionally, breast SWE is demonstrated to be superior to axilla US in detecting residual metastasis in axilla level III after NAT in breast cancer patients with nodal involvement. Furthermore, the combination of breast SWE and axilla US can significantly improve the ability to distinguish negative from positive axilla level III following NAT. These findings provide valuable insights for determining the extent of axillary surgery after NAT in patients with node-positive breast cancer.

According to the guideline of American College of Radiology, US is the most suitable imaging modality for evaluating residual metastasis in the axillary LNs following NAT [11]. However, our study demonstrated that the performance of conventional axilla US is quite limited for diagnosing LNs in axilla level III after NAT in node-positive patients (AUC, 0.67). A fair agreement was observed in the axilla level with positive LNs between axilla US diagnosis and pathologic result from ALND. Particularly in cases with pathologically positive axilla level III, axilla US identified suspicious LNs in axilla level III only in 24.24% of them. Notably, based on ROC curve analysis, the cutoff value of US diagnosis was determined to be a positive axilla level on US of > I for identifying pathologically positive axilla level III after NAT. This means that suspicious LNs in axilla level II or III on US were considered as predictors of pathologically positive axilla level III following NAT. After this modification, the ability of axilla US for detecting residual metastasis in axilla level III improved from 24.24 to 51.52%. However, even if positive axilla level on US of > I was defined as a hint of pathologically positive axilla level III after NAT, approximately half of the cases with pathologically positive axilla level III could not be accurately identified by axilla US. As a result, conventional axilla US evaluation currently does not have the potential to fully replace the surgical staging procedure after NAT.

Metastasis of breast cancer usually progresses from level to level in the axilla region. In contrast to LNs in low-level axilla, diagnosing those in the high-level axilla poses numerous challenges in imaging. Due to the anatomical structure of the axillary region, US waves have poor penetrance in axilla level III, posing a challenge for detecting LNs in axilla level III. Furthermore, LNs in axilla level III often appear as small hypoechoic nodules, making it difficult to distinguish between the nodal medulla and the cortex. Consequently, several conventional US features used to predict LNs malignancy may not be entirely suitable for LNs in axilla level III [22]. The integration of cross-sectional imaging, such as MRI, CT, and PET/CT, offers superior value in visualizing axilla level III and interpectoral LNs, as well as extensive nodal involvement, thus overcoming the constraints of US evaluation, which is frequently confined to axilla levels I and II. However, the overall performance of these imaging modalities for diagnosing axillary LN remains limited, particularly after NAT in breast cancer patients with nodal involvement [23]. Thus, there appears to be a growing need for a more precise and comprehensive role for imaging in determining axillary status after NAT.

Indeed, several studies have suggested the potential of breast cancer stiffness to serve as a predictor of axillary LNs metastasis [24–26]. Liu C et al. demonstrated that higher elasticity in breast cancer corresponded to a higher likelihood of nodal involvement [24]. However, previous studies have primarily focused on the assessment of axillary LNs in breast cancer patients before treatment, and it remains unclear how UE can diagnose axillary LNs after NAT in patients with node-positive breast cancer. In our study, we focused on investigating the extent of axillary LNs with residual metastasis after NAT by using SWE. The findings revealed that SWV values in cases with positive axilla level III after NAT were notably higher than those in cases with negative axilla level III, with SWVmax value emerging as the most significant predictor for axilla level III LNs status. Furthermore, breast SWE significantly outperformed axilla US in detecting residual metastasis in axilla level III with an improved sensitivity of 84.85% and the same specificity of 74.36%. This suggests that the advantage of SWE lies in its ability to identify more cases with positive axilla level III. However, the overall performance of SWE alone remains moderate (AUC, 0.79). The last developed model, combing breast SWE and axilla US, showed significant improvement in the ability to distinguish between negative and positive axilla level III following NAT. In comparison with conventional axilla US alone, the combined model showed an AUC of 0.86, with an obviously enhanced sensitivity of 93.94%, while maintaining a comparable specificity of 75.64%, indicating the great potential of this model in detecting positive LNs in axilla level III.

As the trend toward less aggressive axillary surgery after NAT continues, there is an increasing need for imaging in more comprehensively determining axillary status following NAT. This study represents the first attempt to investigate the value of SWE in determining extent of nodal involvement after NAT in patients with node-positive breast cancer. The developed combined model provides valuable insights for determining the extent of axillary surgery after NAT in patients with confirmed positive LNs. In addition, US is a standard examination for preoperatively assessing breast lesions and axillary LNs in breast cancer patients, and SWE offers the advantages of being cost-effective, nonradiative, and noninvasive. Therefore, the combined model based on US and SWE does not require additional procedures. Moreover, decision curve analysis further suggests its satisfactory clinical utility in facilitating individualized strategies for determining the extent of axillary surgery after NAT.

The present study had several limitations. Firstly, despite being a prospective, multicenter study, the sample size was not large enough, with a small number of cases with positive axilla level III after NAT, which might lead to potential bias. Additionally, in this study, the axilla level III LNs dissection was applied in cases with clinically positive axilla level II/III at initial diagnosis, but we did not perform a subgroup analysis stratified across axilla levels with clinically positive LNs before NAT, which might be confounding factor in determining axilla level with residual metastasis after NAT. Lastly, while cross-sectional imaging modalities like MRI offer superior value in visualizing axilla level III, we did not compare the performance between the developed combined model and these imaging modalities in this regard.

## Conclusion

The overall performance of SWE alone is moderate in detecting residual metastasis in axilla level III after NAT in patients with confirmed nodal involvement. However, SWE outperformed US in identifying the axilla levels with nodal metastasis following NAT. Furthermore, the combination of breast SWE with axilla US showed significant improvement in detecting residual metastasis in the high-level axilla after NAT, compared to convention US alone. This combined approach potentially assists in the decision-making process regarding the appropriate scope of LNs dissection.

## Data Availability

The authors declare that they had full access to all of the data in this study and the authors take complete responsibility for the integrity of the data and the accuracy of the data analysis. Data are available upon reasonable request.

## Abbreviations

ALND: Axillary lymph node dissection
AUC: Area under curve
BUS: B-mode US
ER: Estrogen receptor
HER2: Human epidermal growth factor receptor2
LN: Lymph node
LR: Logistic regression
NAT: Neoadjuvant therapy
Pcr: Pathologic complete response
PR: Progesterone receptor
ROC: Receiver operating characteristic
ROI: Region of interest
SWE: Shear wave elastography
SWV: Shear wave velocity
UE: Ultrasonic elastography
US: Ultrasound

## Declarations

### Guarantor

The scientific guarantor of this publication is Xiao-Qing Pei.

### Registration information

This study was registered on the Chinese Clinical Trial Registry with the registration number ChiCTR2400085035. Additional information about the study can be accessed on the website address http://www.chictr.org/cn/.
